# Novel ACE2 binding in bat merbecoviruses expands potential host range

**DOI:** 10.1371/journal.ppat.1013436

**Published:** 2025-11-11

**Authors:** Xiaoguang Zhang, Xing Ma, Ye Chen, Yabo You, Xin-Xin Li, Jie Chen, Shuhan Kong, Dawei Guo, Xiaoling Li, Michael Veit, Xiaofeng Zhai, Jin Tian

**Affiliations:** 1 Academy for Advanced Interdisciplinary Studies, Engineering Laboratory of Animal Immunity of Jiangsu Province, College of Veterinary Medicine, Nanjing Agricultural University, Nanjing, China; 2 Key Laboratory of Fujian-Taiwan Animal Pathogen Biology, College of Animal Sciences, Fujian Agriculture and Forestry University, Fuzhou, China; 3 College of Life Sciences, Nanjing Agricultural University, Nanjing, China; 4 Institute for Virology, Center for Infection Medicine, Veterinary Faculty, Freie Universität Berlin, Berlin, Germany; 5 State Key Laboratory of Veterinary Biotechnology, Harbin Veterinary Research Institute, Chinese Academy of Agricultural Sciences, Harbin, China; University of Maryland School of Medicine, UNITED STATES OF AMERICA

## Abstract

Coronaviruses often cross species barriers, with receptor binding dictating their host range and zoonotic potential. Merbecoviruses, such as MERS-CoV, typically utilize DPP4 as their receptor, whereas Sarbecoviruses, like SARS-CoV, rely on ACE2. This study explores the receptor usage of four merbecoviruses identified in Vespertilionidae bats: *HKU5*, *BtVs-SC2013*, *HKU25*, and *P. khulii-2011*. Our findings reveal species-specific binding to bat ACE2: *HKU5* binds exclusively to *Pipistrellus abramus* ACE2, *P. khulii-2011* interacts solely with *Murina aurata* ACE2, *BtVs-SC2013* recognizes ACE2 from *Murina aurata* and *Myotis myotis*, and *HKU25* displays the broadest binding range. Beyond bats, *BtVs-SC2013* binds to mink ACE2, while *HKU25* interacts with both mink and pangolin ACE2, hinting at potential intermediate hosts for cross-species transmission. We also elucidated the mechanism behind HKU5’s selective binding preference for P. abramus ACE2. Structural analysis and mutagenesis revealed that a carbohydrate attached at position 329 play a crucial role. Introducing the N-glycosylation site into *P. abramus* ACE2 eliminated binding, while its removal from *P. pipistrellus* ACE2, combined with two additional mutations, restored it. Moreover, we pinpointed key residues in mink ACE2 essential for binding the receptor-binding domain (RBD) of *BtVs-SC2013* and *HKU25*. These findings illuminate the receptor usage and host specificity of bat merbecoviruses, enhancing our understanding of their potential for cross-species transmission and adaptation.

## Author summary

Coronaviruses jumping from bats to humans—sometimes via another animal—can trigger outbreaks or pandemics. To infect a host, a virus must latch onto a receptor, a molecular doorway, on its cells. We studied four bat viruses from the merbecovirus group, related to the Middle East Respiratory Syndrome (MERS) virus, to explore their ability to cross species barriers. Previously thought to bind the DPP4 receptor, we found these viruses actually target ACE2, the same receptor SARS uses. Each virus prefers ACE2 from specific bats, with two also binding to mink and pangolin ACE2—suggesting these animals could bridge the gap to humans. We discovered that sugar molecules on ACE2, especially at one key spot, act like a shield, blocking some viruses from attaching. This research reveals how viruses adapt to new hosts, offering clues about their potential to jump to humans. Understanding these receptor preferences could help predict and prevent future outbreaks.

## Introduction

Coronaviruses (CoVs) are a diverse family of enveloped, single-stranded RNA viruses, known for their broad host range and zoonotic potential. They are classified into four genera—Alphacoronavirus, Betacoronavirus, Gammacoronavirus, and Deltacoronavirus—with Betacoronaviruses encompassing five subgenera, including Sarbeco- and Merbecovirus. Merbecoviruses, exemplified by Middle East Respiratory Syndrome coronavirus (MERS-CoV), have drawn significant scrutiny due to their capacity to breach species barriers, as demonstrated by MERS-CoV’s transmission from dromedary camels to humans, resulting in severe respiratory illness [1,2]. Merbecoviruses are primarily associated with bats as natural reservoirs, notably species within the Vespertilionidae family [3]. Bats are well known for their remarkable diversity and adaptability across various ecological environments. Their significant genetic diversity potentially influences the infection and transmission of these viruses. Increased human-bat interactions driven by human activities, such as hunting and habitat encroachment increase the risk of coronavirus transmission to humans [4]. Previous studies have highlighted the risk of zoonotic spillover and leapfrog transmission of multiple coronaviruses from wildlife and farmed livestock to humans [5–7]. The ecological diversity of bats and their role as viral reservoirs underscore the importance of understanding merbecovirus biology, particularly their mechanisms of host cell entry, which dictate tropism and spillover potential

Entry into host cells by coronaviruses is mediated by the spike (S) protein, which engages specific receptors via its receptor-binding domain (RBD). MERS-CoV and its close relative HKU4 have been shown to engage dipeptidyl peptidase 4 (DPP4) as their primary receptor, leading to an assumption that DPP4 is the universal receptor for merbecoviruses [8–10]. However, recent discoveries have challenged this notion, revealing a broader receptor repertoire within the subgenus. For instance, NeoCoV and PDF-2180, identified in African bat species, utilize angiotensin-converting enzyme 2 (ACE2)—a receptor also exploited by SARS-CoV and SARS-CoV-2—demonstrating an evolutionary flexibility in receptor preference [11].

This shift to ACE2 usage suggests potential parallels with Sarbecoviruses in host range and zoonotic pathways. However, most sequenced coronaviruses remain uncharacterized, posing an unknown risk of spillover to humans due to the lack of isolation and molecular characterization. The receptor usage of many bat merbecoviruses, including HKU5, remains undefined, but previous reports indicate that HKU5, does not bind the ACE2 of *Pipistrellus pipistrellus*, hinting at undiscovered specificity within the group [11]

This study focuses on the receptor usage of four merbecoviruses—HKU5, BtVs-SC2013, HKU25, and P. khulii-2011—identified in Vespertilionidae bats [12–15]. While HKU5 and HKU4 share phylogenetic proximity, their receptor preferences remain incompletely characterized. We aimed to elucidate whether HKU5 employ ACE2 as entry receptor, examining their binding specificity across bat species and potential intermediate hosts like mink and pangolins. By integrating structural analyses, binding assays, and pseudovirus systems, we sought to define the molecular determinants of receptor recognition. This research provides critical insights into the host tropism of bat merbecoviruses, contributing to our understanding of their zoonotic potential and informing surveillance strategies for emerging coronaviruses.

## Results

### Species-specific ACE2 receptor usage of bat merbecoviruses

**Fig 1:**
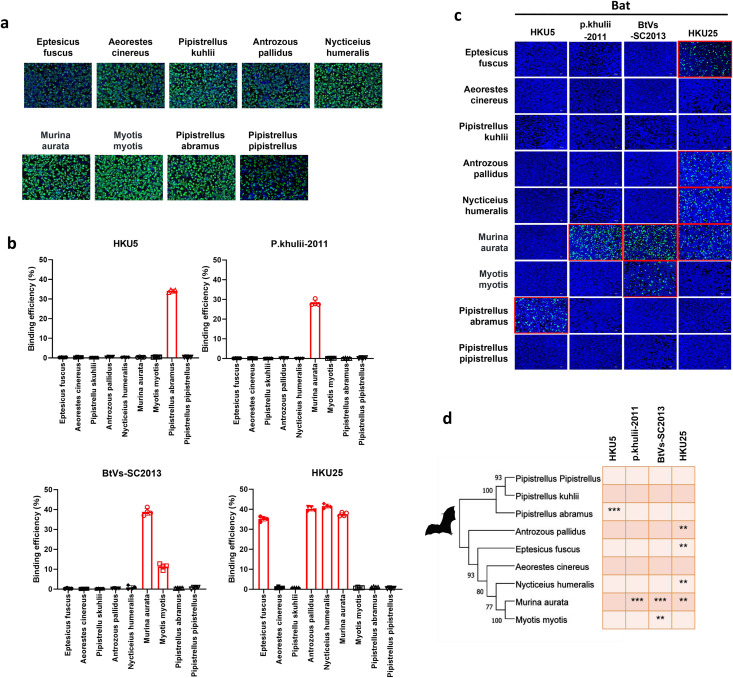
Comprehensive analysis of ACE2 receptor usage by merbecoviruses across bat species. (A) Immunofluorescence assay (IFA) images showing the expression of different bat ACE2 orthologues in cells, Scale bar: 500 μm. (B) RBD-hFc Flow cytometry quantification of the binding of the indicated merbecovirus receptor-binding domain fused with human Fc fragment (RBD-hFc) to cells expressing various bat ACE2 orthologues. (C) Pseudovirus entry efficiency in cells expressing different ACE2 orthologues. (D) Phylogenetic tree of bat species, with the efficiency of merbecovirus pseudovirus entry mediated by their ACE2 orthologues indicated by asterisks: *** for high efficiency, ** for moderate efficiency, and * for low efficiency. The animal material in the picture created with Biorender.

To investigate their ACE2 utilization, we expressed ACE2 from nine bat species on the surface of HEK-293T cells (Fig 1A). Flow cytometry analysis of purified receptor-binding domain fused to human Fc (RBD-hFc) revealed species-specific ACE2 binding patterns for all four viruses. HKU5 specifically bound Pipistrellus abramus (P. abr) ACE2, P. khulii-2011 interacted only with Murina aurata ACE2 and BtVs-SC2013 bound to ACE2 from Murina aurata and Myotis myotis. HKU25 exhibited the most extensive binding spectrum, including Eptesicus fuscus, Antrozous pallidus, Nycticeius humeralis, and Murina aurata ACE2 (Fig 1B). These binding patterns were corroborated using a vesicular stomatitis virus (VSV)-based pseudovirus system that expresses GFP upon cell entry (Fig 1C). This demonstrates the specificity and complexity of Merbecovirus in utilizing bat ACE2 (Fig 1D).

During the revision of this manuscript, the binding of HKU5–19 to P. abr. ACE2 was independently confirmed, and the Cryo-EM structure of the receptor-binding domains (RBD) of the spike protein in complex with P. abr. ACE2 was resolved [16]. Analysis of the 15 interacting residues showed limited conservation across the RBDs of the other bat coronaviruses studied here: eight residues are conserved in HKU25, seven in BtVs-SC2013 and P. khulii-201113–16, and five in PaGB01. Notably, Lys554, which forms the sole ionic bond with Asp329 in P. abr. ACE2, is exclusive to HKU5. The absence of this residue in the other viruses might explain their inability to bind P. abr. ACE2 (Fig A in S1 Appendix).

**Fig 2:**
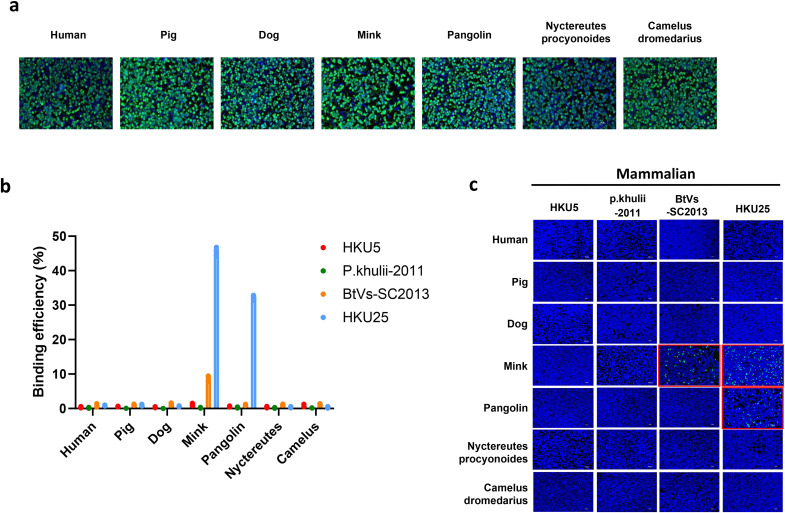
Analysis of ACE2 receptor usage by merbecoviruses across non-bat mammalian species. (A) Immunofluorescence assay (IFA) images showing the expression of different bat ACE2 orthologues in cells, Scale bar: 500 μm (B) Flow cytometry quantification of RBD-hFc binding of the indicated merbecoviruses to various ACE2 orthologues. (C) Pseudovirus entry efficiency in cells expressing different ACE2 orthologues.

We next investigated whether the receptor-binding domains (RBDs) of the four bat merbecoviruses—HKU5, BtVs-SC2013, HKU25, and P. khulii-2011—recognize ACE2 from mammals beyond bats. We expressed ACE2 from putative intermediate hosts (racoon dog, camel, mink and pangolin) and from other mammals (humans, dog and pigs) on the surface of HEK-293T cells (Fig 2A). Using the RBD-hFc binding assay, we identified species-specific ACE2 interactions for two of the viruses. BtVs-SC2013 specifically recognized mink ACE2, while HKU25 bound to both mink and pangolin ACE2. In contrast, HKU5 and P. khulii-2011 showed no binding to any of the ACE2 orthologs tested (Fig 2B). Finally, we used the VSV-based pseudovirus system to corroborate these interactions (Fig 2C). These results suggest that mink and pangolin could serve as potential intermediate hosts for the transmission and adaptation of these merbecoviruses.

### Glycosylation plays a critical role in obstructing viral recognition of bat ACE2 by HKU5

**Fig 3:**
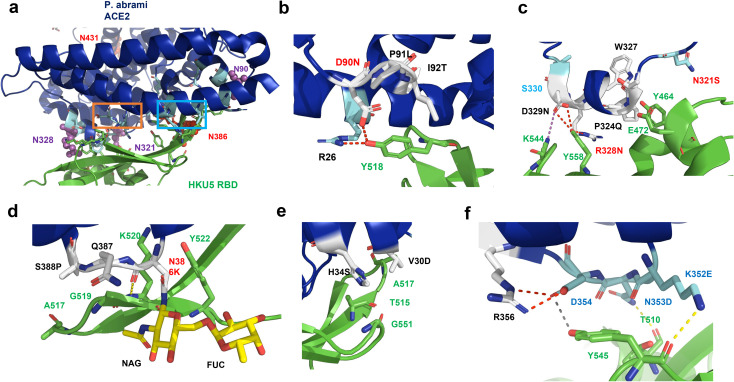
Structure of the HKU5 RBD-ACE2 complex. (A) Structure of the complex between P. abr ACE2 (blue) and the RBD of HKU5 (green). Interacting residues are shown as sticks. N-glycosylation sites on ACE2 near the binding interface, which differ among P. abramus, P. pipistrellus, and P. kuhlii, are shown as spheres: red for sites present in P. abr ACE2 and magenta for sites present in P. pipistrellus and/or P. kuhlii ACE2. (b-e) Detailed views of specific regions: (B) Residues near position 90. (C) Residues near position 321 and 328. (D) Residues near position 386. (E, F) Other residues not involving N-glycosylation sites. The locations of the detailed views of e, f within the overall structure are indicated by cyan and orange frames in panel (A), respectively. The Asn of the N-glycosylation motifs are labelled in red.

Notably, HKU5’s RBD binds Pipistrellus abramus (P. abr.) ACE2 but not those from P. pipistrellus (P. pip.) or P. kuhlii. To elucidate the mechanism behind this selective binding preference for P. abr. ACE2, we conducted a comparative sequence analysis of the 19 ACE2 residues that interact with the HKU5 RBD. This analysis revealed nine differences in P. pip. ACE2 and six in P. kuhlii. ACE2 compared to P. abr. ACE2 (Fig B in S1 Appendix) Strikingly, four differences either introduce or eliminate N-glycosylation sites near the ACE2-RBD interface (Fig 3A). Note that in the N-terminal part of ACE2 from P. abr. one residue is deleted and thus in the other two bat species, residue numbers are shifted by +1. ACE2 of P. pip. contains the N-glycosylation motif _91_NLT^93^ instead of _90_DPI^92^, which might affect contacts with RBD residue Tyr518 (Fig 3B). Similarly, ACE2 from P. pip. and P. kuhlii. feature the sequences _329_NNS^331^ and _329_NKS^331^, respectively, in place of _328_RDS^330^, introducing an N-glycosylation site. The resulting carbohydrate attachment could hinder hydrophilic contacts between Arg328 and Asp329 of ACE2 and Tyr558 and Lys544 of the RBD (Fig 3C). ACE2 from P. abr. and P. kuhlii., but not from P. pip. contain the N-glycosylation motif _321_NMTP^324^ in close vicinity. Here, Pro324 and the residue Tyr327 in close neighborhood form contacts with Glu472 and Tyr 464 of the RBD, which may be influenced by an attached carbohydrate (Fig 3C) Furthermore, P. abr. ACE2 bears a carbohydrate at residue 386 within the sequon _386_NQS^388^, whereas P. pip. and P. kuhlii. ACE2 have the sequence _387_KQP^389^, lacking this glycosylation site. Asn386 establishes main-chain contacts with Lys520 and interacts with Tyr522, while Ser388 engages Ala517 and Gly519 in the RBD (Fig 3D). Other amino acid differences between P. abr ACE2 and ACE2 from the other two bat species not affecting glycosylation include V30D and H34S, which interact with small amino acids in the RBD (Fig 3E) and most notably, in the _352_KND^354^ sequence. Lys352 forms a hydrogen bond with the main-chain oxygen of Tyr545, while Asn353 establishes a hydrogen bond with the side-chain hydroxyl of Thr510 in the P. abr. RBD. Additionally, Asp354 engages in robust interactions with the side chain of Tyr545 (Fig 3F).This sequence is altered to _353_EDD^355^ in P. pip. ACE2 and _353_KDD^355^ in P. kuhlii. ACE2.

**Fig 4:**
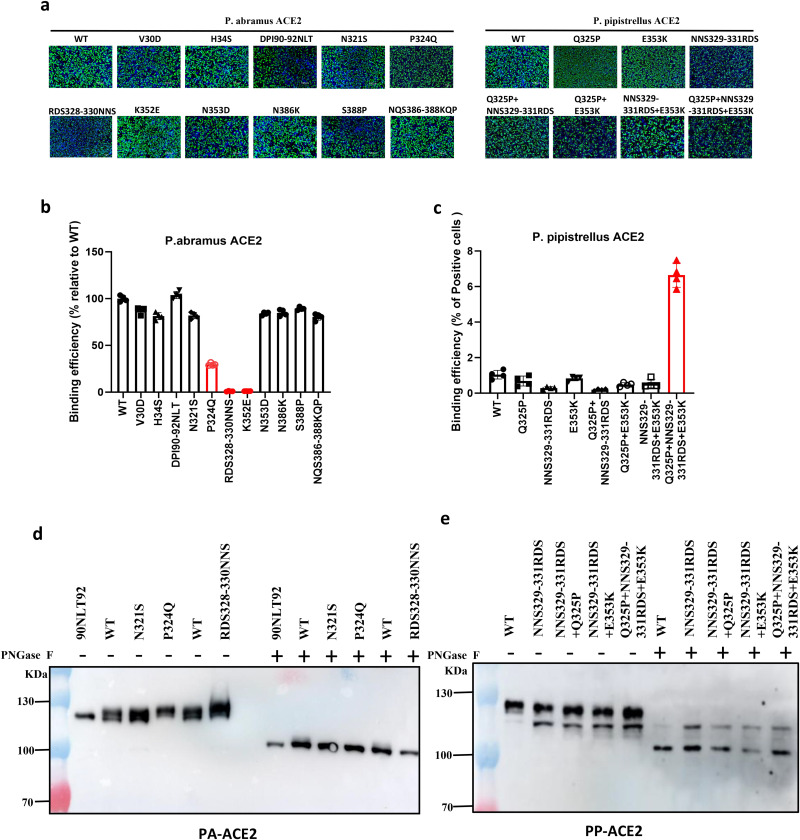
Residues mediating species-specific binding of HKU5 RBD to pipistrellus ACE2. (A) Immunofluorescence assay (IFA) images showing the expression of the indicated mutants of P. abrami and P. pipistrellus ACE2 proteins in cells, Scale bar: 500 μm. (B, C) Flow cytometry quantification HKU5 RBD-hFc binding to wild-type or mutant P. abr. and P. pip. ACE2. All experiments were performed in triplicate. (D, E) Western blot of ACE2 (wild type and the indicated mutants) from P. abramus (PA) (D) and P. pipistrellus (PP) (E) expressed in HEK293T cells. Samples were (+) or were not (-) digested with PNGase F, which removes all N-linked carbohydrates.

To explore the functional implications of these variations, we systematically substituted residues in P. abr. ACE2 with those from P. pip and evaluated their effects on binding of the RBD of HKU5 using live-cell binding assays. Most substitutions, including those introducing (DPI90–92NLT) or eliminating (N321S, N386K) N-glycosylation sites, had minimal impact on HKU5 binding to P. abramus ACE2. However, introducing the N-glycosylation site found in both P. pip. and P. kuhlii. ACE2 through the RDS328–330NNS mutation completely abolished binding. Two additional mutations exhibited significant effects: replacing Lys352, which hydrogen-bonds with the main chain of Tyr 545 in the RBD, with glutamate (K352E) fully disrupted the interaction, whereas P324Q substitution markedly weakened the interaction. Note that substituting Asn353 (N353D) had no effect (Fig 4A, 4B).

To further investigate the determinants of HKU5 RBD binding, we introduced substitutions into P. pip. ACE2, replacing key amino acids with their counterparts from P. abr. ACE2, and assessed whether these changes could enable binding. Individual or paired substitutions—Q325P, E353K, and NNS329–331RDS—failed to confer binding capability to the HKU5 RBD. However, when all three mutations were combined, binding was successfully restored (Fig 4A, 4C).

To determine whether functionally relevant N-glycosylation sites are utilized, we assessed the impact of specific mutations on the SDS-PAGE mobility of ACE2 (Fig 4D). Introducing the glycosylation sequon _90_NLT_92_ into P. abr. ACE2 did not increase its molecular weight, indicating that this site is not glycosylated. Similarly, the _321_NMTP_324_ site in P. abr. ACE2 (mutant N321S) showed no glycosylation, consistent with the absence of a glycan at this position in the cryo-EM structure of P. abr. ACE2 [16]. However, the P324Q mutation increased ACE2 molecular weight, suggesting that the proline adjacent to the N-glycosylation sequon _321_NMT^323^ at position 324 inhibits glycosylation at Asn321 (Fig 4D). This effect is not uncommon, as proline residues are significantly underrepresented at positions 2 and 4 of N-glycosylation sequons [17]. Consequently, while the N321S mutation did not alter HKU5 RBD binding, the P324Q mutation reduced it since a carbohydrate is now attached to N321 (Fig 4B).

We also introduced the P. pip. glycosylation site (RDS329–331NNS) into P. abr. ACE2, which increased its molecular weight relative to the respective wild-type protein (Fig 4D). In contrast, elimination of this glycosylation site from P. pip. ACE2 (NNS329–331RDS) reduced the molecular weight of the protein (Fig 4E). When combined with other mutations, this modification exhibited no further impact on ACE2’s molecular weight (Fig 4E). These findings are consistent with the presence of a carbohydrate at position N329 in P. pip. ACE2 [18].

Thus, susceptibility of P. pipistrellus ACE2 to HKU5 infection depends on removing the glycan at position 329, in conjunction with the E353K and Q325P mutations. Note that the Q325P mutation does not induce glycosylation in P. pip. ACE2, owing to the presence of a serine residue at position 322 (Fig 4E). The negative effect of the Q325P mutation on virus binding (Fig 4C) likely arises from other factors, such as small alterations of the secondary structure of ACE2 by substituting the proline. Furthermore, the E353K substitution facilitates hydrophilic interactions with the main chain at tyrosine 545 in the RBD (Fig 3F).

In P. kuhlii ACE2, which naturally has proline at position 325 and lysine at position 353, the N-glycosylation motif at position 329 likely serves as the primary barrier to HKU5 binding. Eliminating this glycan not only removes a steric hindrance but also enhances interactions between the RBD and residues Arg328 and Asp329 through the NNS329–331RDS substitution (Fig 3C).

### Key mink ACE2 recognition sites for HKU5-related merbecoviruses

**Fig 5:**
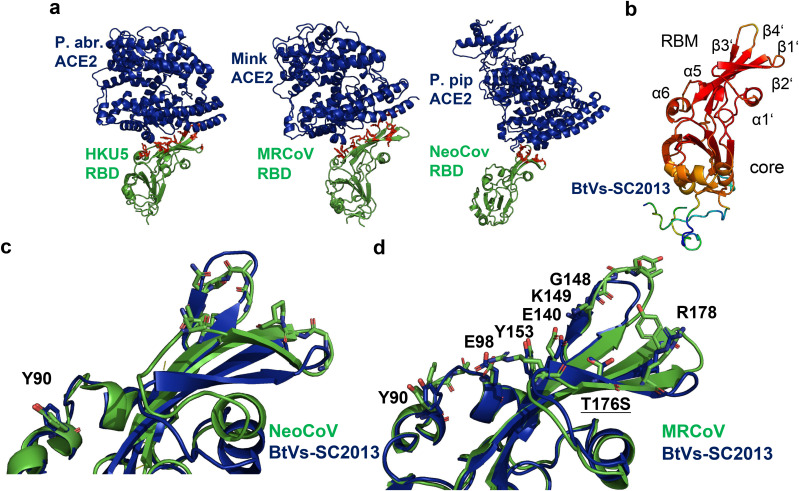
Structural analysis of merbecovirus receptor-binding domains (RBDs) and their interactions with ACE2. (A) Structures of RBD-ACE2 Complexes. Left: RBD of HKU5 bound to ACE2 from P. abrami; (PDB ID: 9D32). Middle: RBD of MRCoV bound to ACE2 from mink (PDB ID: 8ZWE). Right: RBD of NeoCoV bound to ACE2 from P. pipistrellus (PDB ID: 7WPO). (B) Alphafold2 model of the receptor-binding domain (RBD) of BtVs-SC2013, colored by prediction confidence: red (high confidence) to blue (low confidence). (C) Superposition of the RBDs of NeoCoV (green) and BtVs-SC2013 (blue), with a root mean square deviation (RMSD) of 1.037 Å. The 11 residues in NeoCoV that contact ACE2 are depicted as green sticks; among these, only Y90 is identical in BtVs-SC2013. (D) Superposition of the RBDs of MRCoV (green) and BtVs-SC2013 (blue), with an RMSD of 0.76 Å. The 15 residues in MRCoV that interact with mink ACE2 are shown as green sticks. Of these, 7 residues are identical in BtVs-SC2013 (labeled), and one residue, T176 in MRCoV, is replaced by a similar amino acid, S, in BtVs-SC2013. See Fig D in S1 Appendix for AlphaFold2 predictions of the RBDs of HKU25 and Pteropus kuhlii, and Table A in S1 Appendix for amino acid sequence alignments.

We recently isolated a virus closely related to HKU5 from mink, (designated MRCoV), and determined the structure of its RBD bound to the mink ACE2 receptor. While this structure shares overall similarity with HKU5-CoV bound to P. abramus ACE2, both complexes exhibit marked structural divergence from ACE2-bound merbecoviruses PDF-2180 and NeoCoV (Fig 5A) [11].

To further explore the binding modes of the RBDs of BtVs-SC2013 and HKU25, we employed AlphaFold 2 to predict their structures, yielding high-confidence models (pIDDT > 90) that are nearly identical to each other (RMSD: 0.25–0.31 Å) and closely resemble the experimentally determined HKU5 structure (RMSD: 0.54–0.61 Å) (Figs 5B and 5C in S1 Appendix). The receptor-binding domain (RBD) displays the characteristic merbecovirus folding architecture, featuring a β-sheet formed by four β-strands and a single α-helix (α1’), which harbors most of the ACE2-interacting residues. The α5 and α6 helices, integral to the core domain, also contribute residues that facilitate receptor engagement.

A detailed comparative analysis of ACE2-binding residues revealed that BtVs-SC2013, HKU25, and P. khulii-2011 share a higher number of conserved interacting residues with MRCoV (6, 8, and 5, respectively out of 15) compared to PDF-2180 or NeoCoV (1–2 conserved residues out of 10 (PDF-2180) and 11 (NeoCoV)) (Figs 5C, 5D, and 5D in S1 Appendix). This strongly suggests that the bat-dervied merbeoviruses bind to ACE2 in a mode similar to MRCoV.

**Fig 6:**
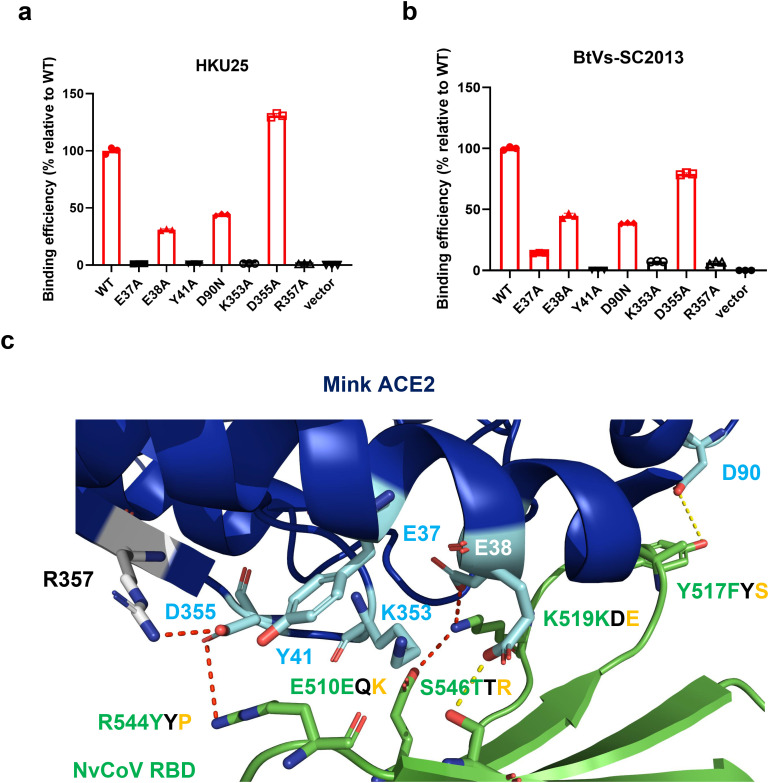
Mode of binding of HKU25 and BtVs-SC2013 RBDs to mink ACE2. (A, B) Flow cytometry quantification of the binding of HKU25 and SC2013 RBD-hFc to wild-type or mutant mink ACE2. (C) Structure of mink ACE2 (blue cartoon) in complex with the RBD of MRCoV (green). Residues in mink ACE2 that were mutated in the binding experiments are highlighted as cyan sticks, while residues in the MRCoV RBD that directly contact ACE2 are shown as green sticks. For each ACE2-contacting residue in MRCoV, the corresponding residues in other RBDs are indicated: green letters indicate residues in HKU25 and BtVs-SC2013 that facilitate binding to mink ACE2, whereas black and orange letters denote residues in Pteropus kuhlii and PaGB01, respectively, that do not support binding.

To validate this hypothesis, we introduced seven mutations into mink ACE2 targeting amino acids known to affect the interactions between mink ACE2 and MRCoV RBD. Six mutations (E37A, E38A, Y41A, D90N, K353A, R357A) substantially disrupted binding to mink ACE2 for the RBDs of both BtVs-SC2013 and HKU25-CoV (Fig 6A, 6B). Notably, only D355A had minimal impact on binding to BtVs-SC2013. This pattern indicates largely conserved but non-identical binding interfaces compared to MRCoV.

To elucidate the differential binding of mink ACE2 by BtVs-SC2013/HKU25 versus P. khulii-2011/PaGB01, we performed residue-level mapping of ACE2-contacting positions in MRCoV RBD (Fig 6C). Sequence alignment revealed complete conservation of these interfacial residues in BtVs-SC2013 and HKU25, with either identical residues or conservative substitutions maintaining physicochemical properties. Conversely, P. kulii and PaGB01 RBDs exhibited non-conservative substitutions at two crucial positions: (i) the K519D/E mutations disrupt the essential salt bridge with mink ACE2 E38 observed in MRCoV complexes. (ii) the E510Q/K substitutions eliminate the complementary salt bridge with K519 required for intra-RBD stabilization.

In summary, our findings highlight a conserved ACE2-binding mode among HKU5-related merbecoviruses, with BtVs-SC2013 and HKU25 closely mirroring the interaction profile of MRCoV, while P. khulii-2011 and PaGB01 diverge due to key interfacial substitutions. These structural and mutational insights underscore the nuanced molecular determinants governing receptor recognition and binding specificity within this viral lineage.

## Discussion

This study reveals unexpected ACE2 receptor usage by four bat-derived merbecoviruses, previously thought to use DPP4. Our findings reveal distinct, species-specific patterns of ACE2 binding among these viruses, all identified in Vespertilionidae bats: HKU5, BtVs-SC2013, HKU25, and P. khulii-2011. Specifically, HKU5 exclusively engages Pipistrellus abramus ACE2, while P. khulii-2011 interacts solely with Murina aurata ACE2. In contrast, BtVs-SC2013 recognizes ACE2 from both Murina aurata and Myotis myotis, and HKU25 exhibits the widest binding repertoire across tested species (Fig 1). A key molecular determinant of this specificity emerged from our analysis: glycosylation differences, particularly at residue 329, act as a critical barrier preventing HKU5 from binding to ACE2 in non-permissive bat species, such as P. pipistrellus and P. kuhlii (Fig 4).

Extending beyond bat hosts, our results highlight broader zoonotic implications. BtVs-SC2013 effectively binds mink ACE2, while HKU25 interacts with both mink and pangolin ACE2 (Fig 2). Further investigation pinpointed specific residues in mink ACE2 that are essential for recognition of the RBD of these merbecoviruses, shedding light on the molecular basis of cross-species compatibility (Fig 5). These findings underscore the adaptability of these viruses and emphasize the importance of understanding receptor tropism in assessing their potential for host-switching events.

While our manuscript was peer-reviewed, several new studies—three published articles and four preprints—have emerged, exploring receptor usage by bat-derived merbecoviruses and providing additional context for our findings. Three independent groups substantiate our observations, confirming that HKU5 binds efficiently to ACE2 from Pipistrellus abramus but shows no detectable interaction with ACE2 from other bat species, such as Pipistrellus pipistrellus [16,19,20]. These results align closely with our data, reinforcing the species-specific receptor preferences we report. Park et al. expand the scope beyond our study, demonstrating that HKU5 also interacts with ACE2 from several non-bat mammalian species, notably within the Bovidae family—an aspect we did not investigate [16]. Similarly, Catanzaro et al. identify P. abramus ACE2 as a functional receptor for HKU5 and report mink ACE2 as the only other effective receptor among those tested. Unlike our approach, which assessed binding using the purified receptor-binding domain fused to human Fc (RBD-hFc) on ACE2-expressing cells, Alfajaro et al. employed pseudoviruses and recombinant HKU5 virus. This methodological difference suggests that binding to mink ACE2 may depend on the full-length HKU5 spike protein, rather than the RBD alone. Notably, both pseudovirus entry and replication of recombinant HKU5 virus were less efficient and slower in cells expressing mink ACE2 compared to those expressing P. abramus ACE2, highlighting potential differences in receptor affinity or downstream entry mechanisms.

Another study identified two merbecoviruses from the European bat species *Pipistrellus nathusii* (P. nat) that utilize ACE2 as their receptor. The RBDs of these viruses also exhibit a restricted host tropism among bats, showing no affinity for ACE2 from other Pipistrellus species, such as P. pipistrellus and P. kuhlii, and limited binding to ACE2 from a few other bat genera. Notably, the RBD of one of these viruses also demonstrates binding to ACE2 from species within the Felidae family, highlighting a broader yet selective receptor usage pattern beyond bats. This study further pinpointed carbohydrates as a key determinant of receptor specificity, noting that a glycan at position 432 impedes efficient binding of these viruses to mammalian ACE2 [21]. Although P. abramus ACE2 is similarly N-glycosylated at this residue, its location outside the interaction interface suggests it is unlikely to influence HKU5 binding (Fig 3A).

None of the viruses examined in these studies bind to human ACE2; however, a few amino acid substitutions in the HKU5 RBD enables this interaction, suggesting a latent potential for adaptation to human receptors [16]. In stark contrast, bat-derived viruses from the HKU5-CoV lineage 2 demonstrate a remarkably broad host tropism, engaging ACE2 from a wide array of bat species as well as numerous non-bat mammals, including humans, and even extending to avian species [22].

One study further delineated the molecular determinants governing HKU5 species tropism, specifically comparing P. abramus and human ACE2, and its findings largely corroborate our own [16]. Among the four key sites identified, two align precisely with our results: (i) residue 324, where a proline is favored, and (ii) residues 328–329, where arginine and aspartate, respectively, are preferred. However, this study did not address aspects uncovered in our work, namely the role of proline at position 324 in preventing N-glycosylation when residue 321 is an asparagine, and the significance of glycosylation at position 328 in P. pipistrellus and P. kuhlii ACE2 as a major barrier to RBD binding. Additionally, the study pinpointed residue 321, favoring tyrosine or histidine, and residue 353, favoring asparagine or arginine, as determinants of species tropism. In our experiments, mutations at these positions (N321S and N353D) yielded no discernible effect, likely due to the substitution of amino acids with similar physicochemical properties. In contrast, our analysis identified residue 352 (mutant K352E) as a critical factor influencing bat species tropism—a site not addressed in the referenced study—highlighting another contribution to the understanding of HKU5 receptor specificity.

Structural analyses accompanying these studies have provided detailed insights into the configurations of the RBDs of these viruses when bound to the ACE2 receptors of their respective hosts [16,20–24]. These investigations unveil distinct binding modes and unique RBD footprints on the ACE2 surface, which not only vary among the viruses studied but also deviate from previously documented structures of ACE2-binding merbecoviruses [11]. This divergence underscores a pattern of convergent evolution, reflecting diverse adaptive strategies for exploiting ACE2 as a receptor. Our own findings further demonstrate that the binding of BtVs-SC2013 and HKU25 to mink ACE2 closely resembles, yet is not identical to, that of MRCoV, reinforcing the concept of nuanced differences in their binding interfaces despite shared receptor usage.

**Fig 7:**
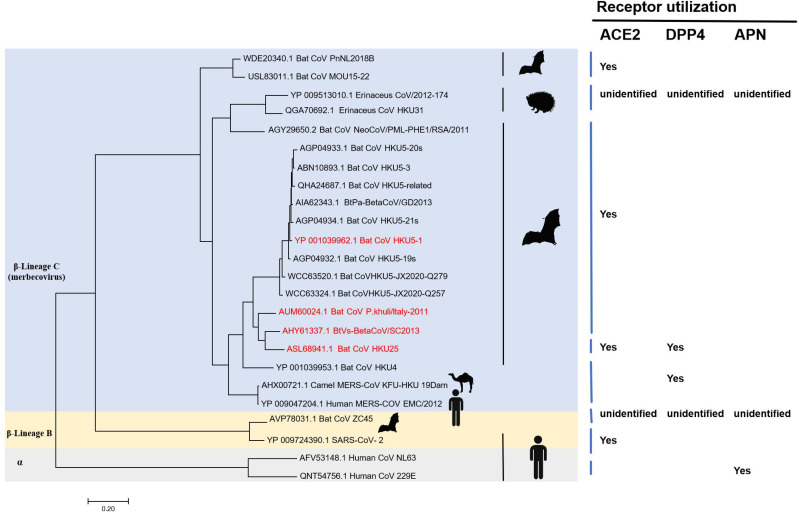
Maximum likelihood phylogenetic tree constructed from full-length spike (S) protein amino acid sequences of merbecoviruses and selected outgroup coronaviruses. Host species are annotated next to each sequence, and receptor usage—ACE2, DPP4 or APN—is indicated. The animal material in the picture created with Biorender.

The maximum likelihood phylogenetic tree, constructed using S protein amino acid sequences, categorized the merbecovirus subgenus into three distinct lineages: A, B, and C. Lineage C forms the largest cluster, comprising viruses from different hosts, including bats, camels, hedgehogs (Erinaceus spp.), and humans. With the exception of HKU4, which utilizes DPP4, and ZC45 from lineage B, whose receptor preference remains unknown, all bat viruses in lineage C employ ACE2 as their primary receptor. This pronounced preference for ACE2 suggests a significant evolutionary adaptation that may enhance the potential of lineage C merbecoviruses to transmit across species, including to humans(Fig 7).

These findings have implications for zoonotic risk assessment. The ability of BtVs-SC2013 and HKU25 to bind mink and pangolin ACE2, combined with their structural similarity to HKU5, suggests a potential for adaptation to new hosts, including humans, if additional mutations occur. Surveillance of potential intermediate hosts like mink, alongside structural studies of viral evolution, will be critical to predict and mitigate future outbreaks. Our study thus contributes to a growing body of evidence that merbecoviruses exhibit remarkable receptor plasticity, warranting further investigation into their ecological and evolutionary dynamics.

## Methods details

### Cells

HEK 293T cells (ATCC with catalog number CRL-3216) were maintained in Dulbecco’s Modified Eagle Medium containing 10% fetal bovine serum (Biological Industries, C04001500) at 37°C, with 5% CO_2_ supplementation. The Expi293F cells (from Thermo Fisher, A14635) were cultured in suspension in Expi293 Expression Medium, at 37°C with 8% CO₂.

### Plasmids

Plasmids expressing bat ACE2 orthologues or mammalian ACE2 from various species [11,25], including Eptesicus fuscus (XP_008153150.2), Aeorestes cinereus (GCA_011751065.1), Pipistrellus kuhlii (XP_036295422.1), Antrozous pallidus (GCA_007922775.1), Nycticeius humeralis (GCA_007922795.1), Murina aurata (GCA_004026665.1), Myotis myotis (XP_036161734.1), Pipistrellus abramus (ACT66266.1), Pipistrellus pipistrellus (GCA_004026625.1), Human (NP_001358344.1), Pig (NP_001116542.1), Dog (NP_001158732.1), Mink (XP_044091953.1), Pangolin (XP_036768816.2), Nyctereutes procyonoides (XP_055195065.1), and Camelus dromedarius (XP_031301717.1), were generated by inserting human codon-optimized sequences into the pCAGGS vector (NovoPro #V008798) such that a Flag-tag (DYKDDDDK) was added to the C-terminus.

The full length, human codon-optimized spike gene of HKU5 (NC_009020.1) or MERS were cloned into the pCAGGS vector. Then MERS RBD sequence was replaced by human codon-optimized RBD sequences of BtVs-SC2013 (KJ473821.1) (342–576 aa), HKU25 (KX442564.1) (375–605 aa) and P. kuhlii 2011 (MG596803.1) (373–604 aa), respectively [11].

The plasmids expressing the recombinant CoVs RBD-hFc fusion proteins were constructed by inserting the coding sequences of the RBDs of HKU5 (375–604 aa), BtVs-SC2013 (381–586 aa), HKU25 (331–524 aa), and P. kuhlii 2011 (481–616 aa) into the pCDNA3.4 vector such that the constant region of a human immunoglobulin G (hFc-tag) was added to the C-terminus.

### Expression and purification of RBD-hFC proteins

Expi293F cells transfected with expression plasmids were cultured for 4 days, the cell culture supernatant was harvested, and incubated overnight at 4°C with protein A agarose (Beyotime, P2015) pre-equilibrated in binding buffer (0.15 M NaCl, 20 mM Na_2_HPO₄, pH 7.0). The supernatant was subsequently removed. The recombinant protein was then carefully eluted from the protein A agarose using a 50 mM glycine buffer adjusted to pH 2.7. Finally, the purified protein was stored at -80°C after exchanging the elution buffer with PBS.

### RBD-hFc live-cell binding assays

Purified coronavirus RBD-hFc recombinant proteins (100 μg/mL) were incubated with HEK 293T cells expressing ACE2 for 1 hour at 37°C at 36 hours post-transfection. After binding, the cells were washed once with Hanks’ Balanced Salt Solution (HBSS) and then incubated with 1 μg/mL of Fluorescein (FITC)–conjugated Affinipure Goat Anti-Human IgG (H + L) (Proteintech, SA00003–12), diluted in HBSS/1% BSA for 1 hour at 37°C. For the indirect immunofluorescence assay (IFA), the cells were washed once with HBSS and then incubated with Hoechst 33342 at 37°C to stain the nuclei. Images were captured using a fluorescence microscope (Nikon). For the flow cytometry analysis, the cells were washed once with HBSS, removed from the plate with trypsin and analyzed using flow cytometry (BD Accuri C6).

### Indirect immunofluorescence assay (IFA)

To validate the expression levels of ACE2 with a C-terminal fused Flag-tag, immunofluorescence assays were conducted. The transfected cells were fixed with 100% methanol, incubated with a mouse anti-Flag M2 antibody (1:500 diluted in PBS, Sigma-Aldrich, catalog number F1804). After washing three times with PBS, the cells were incubated with an Alexa Fluor 488-conjugated goat anti-mouse IgG secondary antibody (1:100 diluted in PBS, Thermo Fisher Scientific, A32742). After three washes with a solution of 0.01% Triton X-100 in PBS, the cells were treated with DAPI (Solarbio, used at a 1:1000 ratio) for 10 minutes. Finally, the preparations were observed under a fluorescence microscope manufactured by Nikon.

### Production of pseudotyped virus particles

Pseudotyped viruses bearing coronavirus spike protein (CoV-PSVs) were generated using a replication-deficient VSV (vesicular stomatitis virus) pseudotyping system (VSV-dG). To generate CoV-PSVs, the HEK293T cells were transfected with plasmids encoding coronavirus spike protein. Following 24–36 hours of transfection, the cells were infected with VSV-dG-GFP (MOI = 1) diluted in DMEM containing 8 μg/ml polybrene at 37°C for 4 hours. The virus inoculum was removed and the cells were washed five with PBS. To minimize the background from the parental virus, the medium was replaced with DMEM containing the low density lipoprotein receptor ligand-binding domain (LDLR-LBD) protein, which is a receptor for VSV. After 24 hours, the resulting CoV-PSV-containing supernatant was removed and stored at −80°C. The titer of the pseudotyped viruses was then evaluated by determining the TCID_50_ using the Reed-Muench method. HEK293T cells expressing Pipistrellus abramus-ACE2 were used for titration of the HKU5 S pseudotyped viruses, and HEK293T cells expressing Murina aurata-ACE2 were also used for titration of P. kuhlii 2011, BtVs-SC2013 and HKU25 S pseudotyped viruses.

### Infection assay

Pseudotyped virus particles were employed to infect HEK 293T cells that overexpressed bat or mammalian ACE2 orthologs. The specific MOI (multiplicity of infection) was 5 for HKU5, Pipistrellus kuhlii 2011, and BtVs-SC2013 strains and 1 for HKU25. To assess the infection efficiency, data on EGFP expression was recorded 36 hours post-infection using a high-resolution fluorescence microscope made by Nikon.

### Analysis of the usage of N-glycosylation sites in bat ACE2

The plasmid, expressing either wild type or mutant ACE2, fused to a C-terminal Flag-tag, was transfected into HEK 293T cells. After 24-hours, the cell supernatant was removed, and each well was lysed using 200 μl of NP40 lysis buffer supplied by NEB. Subsequently, after centrifugation, the resulting supernatant was transferred to a fresh centrifuge tube, then digested with PNGase F (Yeasen, 20407ES01) according to the manufacturer’s instructions. The molecular weights of the resulting proteins were determined by SDS-PAGE and Western Blot with a mouse anti-Flag M2 antibody (1:500 diluted in PBST, Sigma-Aldrich, catalog number F1804).

### Alphafold predictions

The colab version of AlphaFold 2 (https://colab.research.google.com/github/sokrypton/ColabFold/blob/main/AlphaFold2.ipynb#scrollTo=ADDuaolKmjGW) was used with the following settings: “use_templates”: true, “num_relax”: 1, “relax_max_iterations”: 200, “relax_tolerance”: 2.39, “msa_mode”: “mmseqs2_uniref_env”, “model_type”: “AlphaFold 2_ptm”, “num_models”: 5, “num_recycles”: 3, “rank_by”: “plddt”,”pair_mode”: “unpaired_paired”, “pairing_strategy”: “greedy”, “random_seed”: 0, “num_seeds”: 1. The predicted structures were analyzed with PyMol (Version 2.1.1, Schrodinger LLC).

### Sequences alignment and phylogenetic tree construction

Phylogenetic analysis of merbecoviruses was conducted using the full-length S amino acid sequence with additional sequences from other coronaviruses included as outgroups. The sequences were retrieved from the NCBI database and aligned using Clustal Omega.

A maximum likelihood phylogenetic tree was constructed using MEGA 12, employing the Jones-Taylor-Thornton (JTT) model of amino acid substitution and 1000 bootstrap replicates. The sequences are as follows: PaGB01 (WDQ26963.1) PnNL2018B (WDE20340.1), MOU15–22 (USL83011.1), Erinaceus CoV/2012–174 (YP 009513010.1), Erinaceus CoV HKU31 (QGA70692.1), NeoCoV/PML-PHE1/RSA/2011 (AGY29650.2), Bat CoV HKU5-20s (AGP04933.1), Bat CoV HKU5–3 (ABN10893.1), Bat CoV HKU5-related (QHA24687.1), BtPa-Beta CoV/GD2013 (AIA62343.1), Bat CoV HKU5-21s (AGP04934.1), Bat CoV HKU5–1 (YP 001039962.1), Bat CoV HKU5-19s (AGP04932.1), Bat CoV HKU5-JX2020-Q279 (WCC63520.1), HKU5-JX2020-Q257 (WCC63324.1), Bat CoV P.khuli/ltaly-2011 (AUM60024.1), BtVs-Beta CoV/SC2013 (AHY61337.1), Bat CoV HKU25 (ASL68941.1), Bat CoV HKU4 (YP 001039953.1), Camel MERS-CoV KFU-HKU19Dam (AHX00721.1), Human MERS-CoV EMC/2012 (YP 009047204.1), Bat CoV ZC45 (AVP78031.1), SARS-CoV- 2 (YP 009724390.1), Human CoV NL63 (AFV53148.1), Human CoV229E (QNT54756.1).

### Statistical analysis

Experiments were performed 2–3 times, each with 3 or 4 biological replicates. Representative results are presented. Data are expressed as mean ± SD or mean ± SEM, as indicated in the figure legends. All statistical analyses were performed using unpaired two-tailed t-tests in GraphPad Prism 9.

## Supporting information

S1 Appendix Including: Fig A. Sequence alignment of receptor-binding domains (RBDs) from HKU5 and other merbecoviruses, highlighting conservation of ACE2-contacting residues. The RBD sequence of HKU5 was obtained from its crystal structure in complex with Angiotensin-Converting Enzyme 2 (ACE2) of Pipistrellus abramus (PDB ID: 9D32). This sequence was aligned with the corresponding RBD sequences of PaGB01, BtVs-SC2013, HKU25, and P. kuhlii-2011 using Clustal Omega. In the alignment: Residues of HKU5 that contact P. abramus ACE2 are highlighted in yellow, with the residue involved in ionic interactions bolded and underlined. Residues that are identical to the HKU5 interacting residues are highlighted in green in each RBD sequence. Only the regions of the RBD sequences involved in ACE2 interaction are displayed.
(PDF)
