# Supplementary material for: Novel ACE2 binding in bat merbecoviruses expands potential host range
Source: PLoS Pathog. 2025 Nov 11;21(11):e1013436. doi: 10.1371/journal.ppat.1013436 (PMC12604803; doi:10.1371/journal.ppat.1013436)
Supplement: S1 Appendix — Sequence alignment of receptor-binding domains (RBDs) from HKU5 and other merbecoviruses, highlighting conservation of ACE2-contacting residues. The RBD sequence of HKU5 was obtained from its crystal structure in complex with Angiotensin-Converting Enzyme 2 (ACE2) of Pipistrellus abramus (PDB ID: 9D32). This sequence was aligned with the corresponding RBD sequences of PaGB01, BtVs-SC2013, HKU25, and P. kuhlii-2011 using Clustal Omega. In the alignment: Residues of HKU5 that contact P. abramus ACE2 are highlighted in yellow, with the residue involved in ionic interactions bolded and underlined. Residues that are identical to the HKU5 interacting residues are highlighted in green in each RBD sequence. Only the regions of the RBD sequences involved in ACE2 interaction are displayed. (PDF) [file ppat.1013436.s001.pdf]

S1 Fig

```
                           461 464         472
HKU5            LTVDYFAYSTDMSSYLQPGSAGEIVQFNYKQDFSNPTCRVLATVPQNLTT---ITKPSNY  506
PaGB01          LTVDYFAYPSSMASYLQKGSTGEIAQFNYKQDFTNPTCRILATVPSNVT----IHKPDDY  505
BtVs-SC2013     LTVDYFAYPTYMSSYLQQGSTGEISQFNYKQDFSNPTCRILATVPANLSASGLLPKPSNY  509
HKU25           LTVDYFAYPLSLASYLQQGSTGEITQYNYKQDFSNPTCRILATAPANIT----LTKPNNY  505
p.Kulii-2011    LTVDYFAYPLYLASYLQQGSTGEIAQYNYKQDFSNPTCRILASVPANVS----IPKPDKY  505
                ********   ::**** * :*** ::******:.****:**:.* *::    : **..*

                   510     515 518 520                  544
HKU5            AYLTECYKTSAY-GKNYLYNAPGGYTPCLSLASRGFSTKYQSHS----DGELTTTGYIYP  561
PaGB01          RWLTKCYTLTS-KGEVLHYVEPGQYTPCLTLFSHQNNNDPDRLSATANT--MA-------  556
BtVs-SC2013     VWLSECYQNS-FTGKNFQYVKAGQYTPCLGLAANGFEKSYQTHRDPVSK—LAVTGVVTP   563
HKU25           NWLTECYHSTAF-GKQPYYVQPGQYTPCLGLAISGFTTSYETRRDPNTK—MAATGLVTA   563
p.Kulii-2011    IWLSQCYSFSAYSGDIPHYVQPGQYTPCLYLTSSGFDKSYQTNRDMQNK—MAATGVTSS   564
                  *::**  :   *.   *   * ***** *      .. :          :.
```

S2 Fig

```
                                      26  30 33/4
Bat-PA      MSSSSWLLLSLVAVAGAQY-TTEEEARRFLVKFNHEAENLSHESALASWDYNTNITDENA   59
Bat-PK      MSGSFWLFLSLVAVAAAQSSTTEGEAREFLDKFNSKAENLSHESALASWDYNTNITDKNA   60
Bat-PP      MSGSFWLFLSLVAVAAAQSRTTEEEARDFLDKFNSEAENLSHESALASWDYNTNITDENA   60
            * .* **:***.*::.**  ***  *: ** *** :**:**::.:****:*******:*

                                          90 93 96
Bat-PA      KKMNEADNKWSDFYKEQSKIAQGFPLQEIKDPIIKLQLQILQQNGSSVLTAEKRKRLSTI   119
Bat-PK      QKMNEADSKWSDFYKEQSKLAQAFPLQEIQDSTIKLQLQILQQNGSSVLTAEKSKRLSTI   120
Bat-PP      QKMNEADSKWSDFYKEQSKRAQAFPLQEIQNLTIKLQLQILQQNGSSVLTAEKSKRLSTI   120
            :*** *. *** **:*:*: *: :**:**::  ** **: ***.*****:*:* :**.**

                                    323 327                      352
Bat-PA      QSWDAEKIFKEAEKFYSSVGLPNMTPGFWRDSMLTEPSDGRQVVCHPTAWDLGKNDFRIK   358
Bat-PK      QSWDADKIFKEAEKFYLSVGLRNMTPGFWNKSMLTEPSDGRQVVCHPTAWDLGKDDFRIK   359
Bat-PP      QSWDAEKIFKEAEKFYLSVGLYSMTQGFWNNSMLTEPSDGRQVVCHPTAWDLGEDDFRIK   359
            ***** :********: **** .** ***..******.*.*:***********:.*****

                                      385 388  392
Bat-PA      MCTKVTMDDFLTAHHEMGHIQYDMAYANQSYLLRNGANEGFHEAVGEVMSLSVATPKHLK   418
Bat-PK      MCTKVTMDDFLTAHHEMGHIQYDMAYAKQPYLLRNGANEGFHEAVGEVMSLSVATPKHLK   419
Bat-PP      MCTKVTMDDFLTAHHEMGHIQYDMAYAKQPYLLRNGANEGFHEAVGEVMSLSVATPKHLK   419
            *************************** * :****************:****.***:***
```

S3 Fig

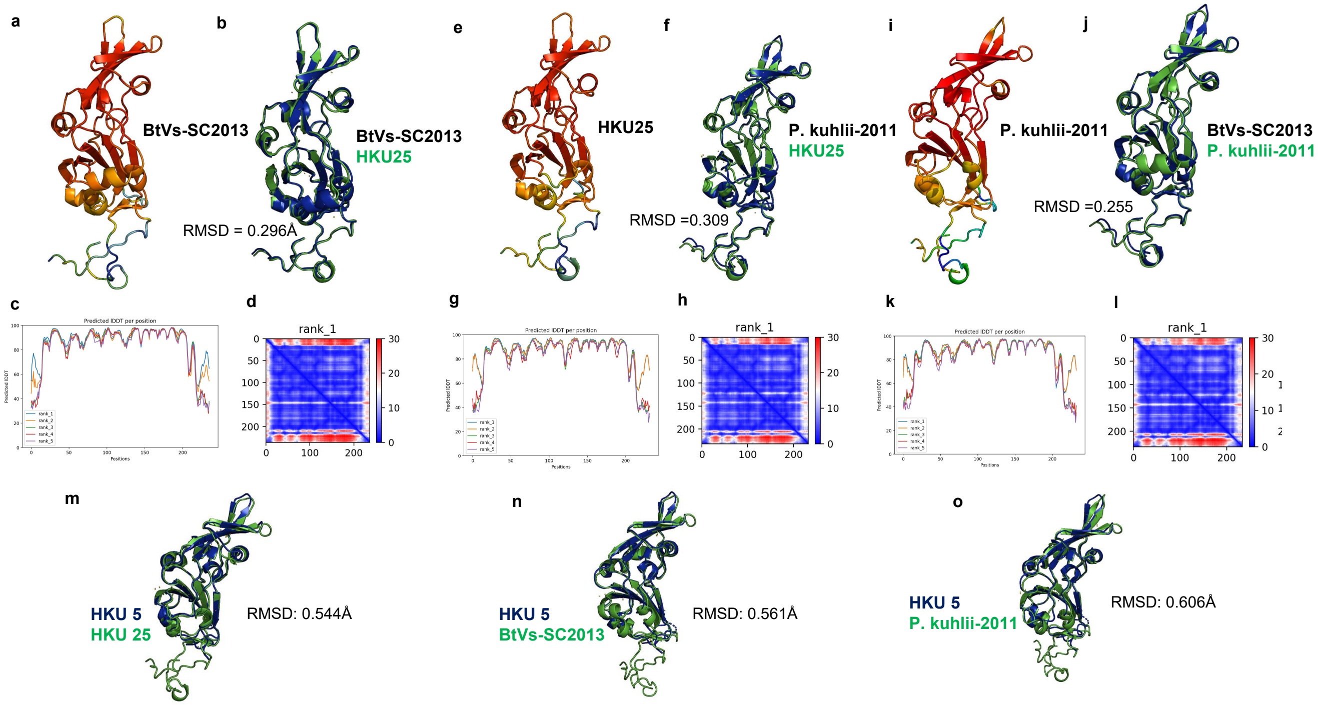

a
b
e
f
i
j
BtVs-SC2013
BtVs-SC2013
HKU25
HKU25
P. kuhlii-2011
HKU25
P. kuhlii-2011
BtVs-SC2013
P. kuhlii-2011
RMSD = 0.296Å
RMSD =0.309
RMSD =0.255
c
d
g
h
k
l
Predicted lDDT per position
rank_1
m
n
o
HKU 5
HKU 25
RMSD: 0.544Å
HKU 5
BtVs-SC2013
RMSD: 0.561Å
HKU 5
P. kuhlii-2011
RMSD: 0.606Å

S4 Fig

```
PDF-2180       FPYAWKSYLAIGSADRIVRFNYNQDYSNP--RIHSKVNSSVG----ISYSGLYSYITNCN    54
NeoCoV         FPYAWKSYLAIGSADRIVRFNYNQDYSNPSCRIHSKVNSSIG----ISYAGAYSYITNCN    56
P.Kulii-2011   YPLYLASYLQQGSTGEIAQYNYKQDFSNPTCRILASVPANVS----IPKPDKYIWLSQCY    56
HKU25          YPLSLASYLQQGSTGEITQYNYKQDFSNPTCRILATAPANIT----LTKPNNYNWLTECY    56
BtVs-SC2013    YPTYMSSYLQQGSTGEISQFNYKQDFSNPTCRILATVPANLSASGLLPKPSNYVWLSECY    60
               :*    ***  **:..* ::**:**:***  ** :.. .:     :   . * ::::*

PDF-2180       YGGFNK---DDVVKPGGRASQPCVTGALNSPTNGQVWSFNFGGVPYRTSRLTYTDH----    107
NeoCoV         YGATNK---DDVVKPGGRASQQCITGALNSPTTGQLWAYNFGGVPYRVSRLTYTDH----    109
P.Kulii-2011   SFSAYSGDIPHYVQPGQ--YTPCLYLTSSG----FDKSYQTNRDM--QNKMAATGVTSS-    107
HKU25          HST-AFGKQPYYVQPGQ--YTPCLGLAISG----FTTSYETRRDP--NTKMAATGLVTAM    107
BtVs-SC2013    QNS-FTGKNFQYVKAGQ--YTPCLGLAANG----FEKSYQTHRDP--VSKLAVTGVVTPM    111
                           *: *      *:  : ..      :::        .::: *.

PDF-2180       --- 107
NeoCoV         --- 109
P.Kulii-2011   --- 107
HKU25          T-- 108
BtVs-SC2013    TSA 114
```

```
#  Percent Identity  Matrix - created by Clustal2.1
     1: PDF-218       100.00   88.79   28.28   28.57   32.65
     2: NeoCoV         88.79  100.00   26.73   29.00   33.00
     3: P.Kulii-2011  28.28    26.73  100.00   66.98   66.04
     4: HKU25_1        28.57   29.00   66.98  100.00   67.59
     5: SC2013         32.65   33.00   66.04   67.59  100.00
```

```
CLUSTAL O(1.2.4) multiple sequence alignment

batHKU5        YSTDMSSYLQPGSAGAIVQFNYKQDFSNPTCRVLATVPQNLTT---ITKPSNYAYLTECY    57
MRCoV          YPTSMSSYLQPGFAGEIVKFNYKQDFSSPTCRVLATVPSNLTT---ITKPSNYVHLTECY    57
P.Kulii-2011   YPLYLASYLQQGSTGEIAQYNYKQDFSNPTCRILASVPANVS----IPKPDKYIWLSQCY    56
HKU25          YPLSLASYLQQGSTGEITQYNYKQDFSNPTCRILATAPANIT----LTKPNNYNWLTECY    56
BtVs-SC2013    YPTYMSSYLQQGSTGEISQFNYKQDFSNPTCRILATVPANLSASGLLPKPSNYVWLSECY    60
               *   ::**** * :* * ::*******.****:**:.* *::     : **.:*  *::**

batHKU5        KTS-AYGKNYLYNAPGAYTPCLSLASRGFSTKYQSHS----DGELTTTGYIYP----      105
MRCoV          KGT-AYGKNYLYNAPGGYTPCLSLASSGFSSDRQSHRQQLSDGYLVTTGSVYA----      109
P.Kulii-2011   SFSAYSGDIPHYVQPGQYTPCLYLTSSGFDKSYQTNRDMQNK--MAATGVTSS----      107
HKU25          HST-AFGKQPYYVQPGQYTPCLGLAISGFTTSYETRRDPNTK--MAATGLVTAMT--      108
BtVs-SC2013    QNS-FTGKNFQYVKAGQYTPCLGLAANGFEKSYQTHRDPVSK--LAVTGVVTPMTSA      114
                 :   *.    *   * ***** *:  **  .. ::.      .  :..**
```

```
#  Percent Identity  Matrix - created by Clustal2.1

     1: MRCoV         100.00   79.05   48.11   56.60   59.81
     2: batHKU5        79.05  100.00   50.98   55.88   62.14
     3: P.Kulii-2011  48.11    50.98  100.00   66.98   66.04
     4: HKU25_1        56.60   55.88   66.98  100.00   67.59
     5: BtVs-SC2013   59.81    62.14   66.04   67.59  100.00
```

S1 Table

S1 Table. Residues of NvHKU5r-CoV RBD interacting with minkACE2 at the binding interface (using a cutoff distance of 4.0Å)

NvHKU5r-CoV RBD	minkACE2
Met460	Gln329
Tyr463	Trp328
Phe468	Asn322
Tyr463<br>Phe468<br>Gly470<br>Glu471<br>Ile472	Glu325
Glu510<br>Arg544<br>Ser546	Lys353
Thr515<br>Ala516<br>Gly554<br>Tyr555	Glu30
Ala516<br>Tyr517	Lys26
Ala516	Gln96
Ala516<br>Tyr517<br>Gly518	Pro389
Tyr517	Asp90
Gly518	Gln388
Lys519	Glu37<br>Arg393
Lys519<br>Tyr521	Ala386
Lys519<br>Tyr521	Ala387
Arg544	Tyr41<br>Gly326<br>Asn330<br>Asp355
Ser546	Glu38
Arg548<br>Val557	Tyr34
Tyr555	Asn33
Tyr521<br>Arg544<br>Glu510	His354
